# Isolated Cardiac Sarcoidosis Presenting as Torsades de Pointes in a Patient With Non-ischemic Cardiomyopathy: A Case Report

**DOI:** 10.7759/cureus.29067

**Published:** 2022-09-12

**Authors:** Moustafa S Alhamadh, Thamer S Alhowaish, Abdulrahman Yousef Alhabeeb, Rakan B Alanazi, Ayah Boudal, Khalid Al Khathlan, Abdulrahman Alrashid

**Affiliations:** 1 Internal Medicine, College of Medicine, King Saud bin Abdulaziz University for Health Sciences, King Abdullah International Medical Research Center, Ministry of the National Guard-Health Affairs, Riyadh, SAU; 2 Neurology, College of Medicine, King Saud bin Abdulaziz University for Health Sciences, King Abdullah International Medical Research Center, Ministry of the National Guard-Health Affairs, Riyadh, SAU; 3 Orthopedic Surgery, College of Medicine, King Saud bin Abdulaziz University for Health Sciences, King Abdullah International Medical Research Center, Ministry of the National Guard-Health Affairs, Riyadh, SAU; 4 Medicine, College of Medicine, King Saud Bin Abdulaziz University for Health Sciences, Riyadh, SAU; 5 Rheumatology, Department of Medicine, Division of Rheumatology, Ministry of Health, Jeddah, SAU; 6 Rheumatology, Department of Medicine, Division of Rheumatology, Ministry of the National Guard-Health Affairs, Riyadh, SAU; 7 Rheumatology, Department of Medicine, Division of Rheumatology, King Abdullah International Medical Research Center, Ministry of the National Guard-Health Affairs, Riyadh, SAU

**Keywords:** cardiac positron emission tomography, cardiac magnetic resonance imaging, torsades de pointes, extrapulmonary sarcoidosis, cardiac sarcoidosis

## Abstract

Sarcoidosis is an immune-mediated, inflammatory, non-caseating-granulomatous disease that can virtually infiltrate any organ. Cardiac sarcoidosis is a leading cause of death in patients with sarcoidosis. Its clinical presentation is highly heterogenous and unpredictable, ranging from asymptomatic to life-threatening conduction disturbances, such as ventricular arrhythmias, and heart failure. Herein, we report a case of isolated cardiac sarcoidosis presenting as sinus bradycardia with first-degree atrioventricular block and an episode of non-sustained polymorphic ventricular tachycardia in a 42-year-old male with non-ischemic cardiomyopathy. He was diagnosed by cardiac magnetic resonance imaging and positron emission tomography with fluorodeoxyglucose and treated with oral prednisone.

## Introduction

Sarcoidosis is an immune-mediated, chronic, inflammatory, non-caseating granulomatous disease characterized by CD4 helper T-cells response to an unknown antigen, which if left untreated, causes progressive fibrosis and end-organ damage [1,2]. African Americans and Scandinavians have the highest incidence of sarcoidosis, with almost three-quarters of the cases occurring in patients aged 25-40 years and an estimated lifetime risk of 0.85% for Caucasian Americans and 2.4% for African Americans [3]. Although the majority of sarcoidosis cases involve the pulmonary system, it can infiltrate the skin, nervous system, musculoskeletal, liver, and heart [4]. Cardiac sarcoidosis (CS) represents 5-10% and, together with neurosarcoidosis, is one of the leading causes of death in patients with sarcoidosis [4,5]. The clinical presentation of CS is highly variable, ranging from asymptomatic to life-threatening conduction disturbances, such as atrial or ventricular arrhythmias, and heart failure [4]. Historically, the diagnosis of CS required endomyocardial biopsy, but due to its low sensitivity rate of 20-30% and invasiveness, it has been largely replaced by cardiac magnetic resonance imaging (CMR) and positron emission tomography (PET) with 18F-fluorodeoxyglucose (FDG) [6]. Corticosteroid is the mainstay of therapy for CS, and prompt recognition and treatment are essential to decrease CS morbidity and mortality. In addition, some patients might need antiarrhythmic medications or catheter ablation and an implantable cardioverter-defibrillator (ICD). Most patients respond well to steroids, however, for refractory CS, immunosuppressants such as methotrexate can be used as a second-line treatment [2,7].

## Case presentation

A 42-year-old male, a known case of non-ischemic cardiomyopathy with reduced ejection fraction (EF), presented to our emergency department due to a recent device shock and a week-long history of recurrent dizziness, lightheadedness, and near-fall episodes. He denied a history of chest pain, palpitation, syncope, shortness of breath, cough, fever, chills, abdominal pain, or nausea and vomiting. He was an active tobacco smoker and had a remote history of performance-enhancing drugs and anabolic steroid use for bodybuilding. Upon admission, he was on multiple anti-failure medications, including sacubitril-valsartan, dapagliflozin, ranolazine, bisoprolol, and spironolactone, and had an ICD implanted a year ago. His family history was noncontributory. On examination, the patient was vitally stable and exhibited no signs of distress. The patient’s chest was clear with equal bilateral air entry and normal vesicular breathing. Cardiovascular examination was unremarkable with normal S1 and S2, no jugular vein distention, lower limbs edema, added sounds or murmurs, carotid or femoral bruits, and thrills or heaves. His abdomen was nontender, soft, and without evidence of organomegaly. He was admitted under cardiology for observation and management as appropriate.

Laboratory investigations

Laboratory values were within normal limits, except for elevated BNP (52.7 pmol/L), mildly elevated glutamic acid (67 umol/L), and low high-density lipoprotein (HDL) (0.82 mmol/L). The rest of the laboratory investigations, including complete blood count with differential, coagulation profile, lipid profile, renal, liver, and thyroid function tests, electrolytes, inflammatory markers, luteinizing hormone (LH), follicle-stimulating hormone (FSH), amino acids, testosterone level, angiotensin-converting enzyme (ACE) level, cardiac biomarkers, and urinalysis were unremarkable.

Imaging

**Figure 1:**
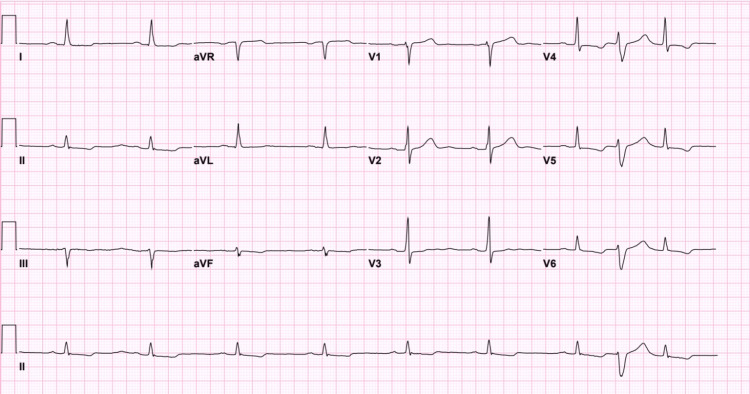
ECG showing sinus bradycardia with first-degree atrioventricular block and frequent premature ventricular complexes

Chest radiograph was unremarkable, and admission ECG showed sinus bradycardia, with first-degree atrioventricular block and frequent premature ventricular complexes (PVCs), and was consistent throughout the patient’s admission (Figure 1).

ICD device interrogation revealed non-sustained polymorphic ventricular tachycardia and an episode of ventricular fibrillation. Findings of transthoracic echocardiogram were mild mitral regurgitation, a severely dilated left ventricle, mild eccentric hypertrophy, global hypokinesis with an EF of 35-30%, and grade 1 diastolic dysfunction. The right ventricle was moderately dilated with mildly reduced systolic function (Video 1).

**Video 1:** Transthoracic echocardiogram showing a dilated left ventricle with mild eccentric hypertrophy.

**Figure 2:**
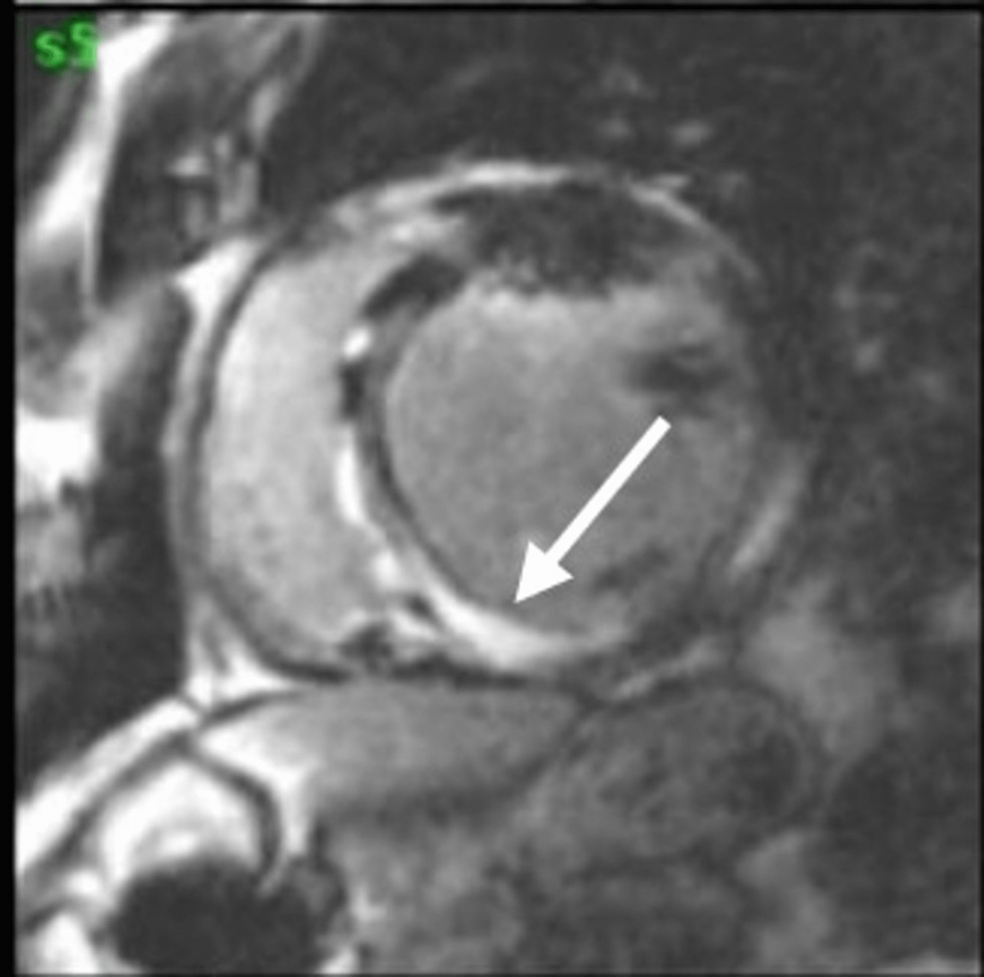
CMR showing a transmural delayed enhancement in the inferior wall and septum with a focal area of transmural delayed enhancement in the apical segment of the anterior wall The white arrow indicates the delayed enhancement in the inferior wall and septum. CMR: cardiac magnetic resonance

Twenty-four-hour cardiac monitoring revealed an average heart rate of 57 bpm and very frequent PVCs with triplets, couplets, bigeminy, and trigeminy patterns, and an episode of non-sustained polymorphic ventricular tachycardia of 150 bpm. The patient underwent CMR, which showed a transmural delayed gadolinium enhancement in the inferior septum and inferior wall with an area of microvascular obstruction, indicating an acute insult. Additionally, there were small areas of delayed gadolinium enhancement in the anterior septum and the subepicardium of the mid-segment of the inferior wall, highly suggestive of CS (Figure 2).

**Figure 3:**
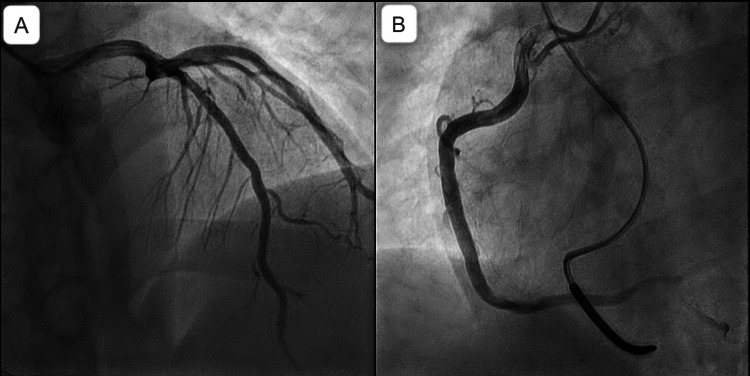
Coronary angiography showing normal and patent coronaries A: Patent left coronary artery B: Patent right coronary artery

Diagnostic coronary angiography was performed but did not identify any abnormality (Figure 3).

**Figure 4:**
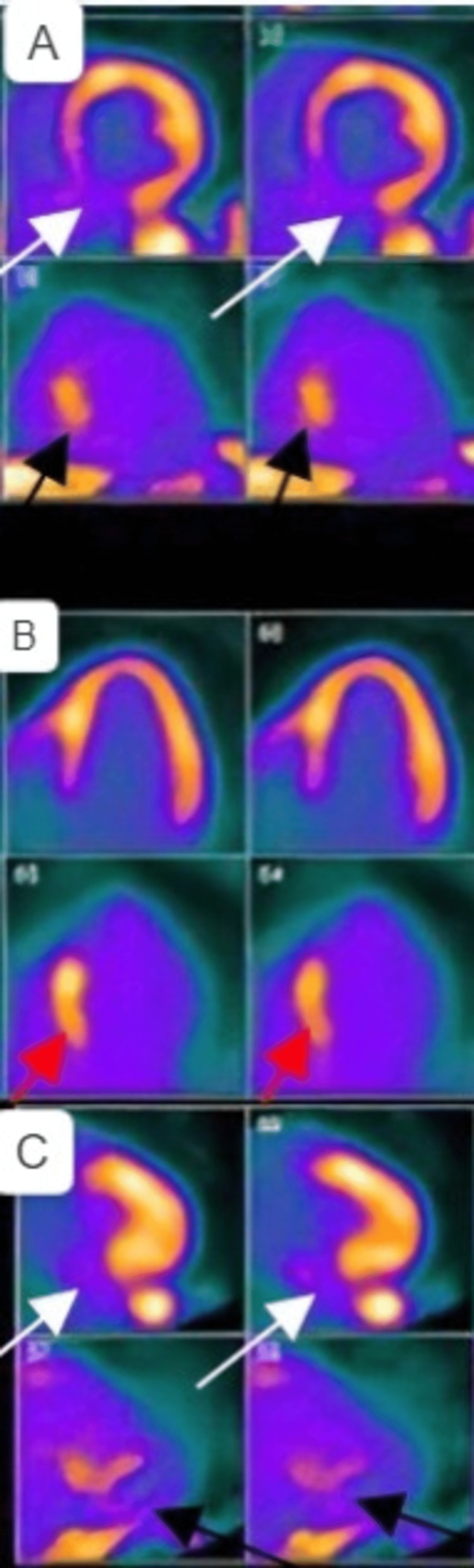
PET/CT myocardial perfusion scan showing a severe perfusion defect in the basal segment of the inferior septum A: The white arrows indicate a moderate-size rest perfusion defect in the inferior septal and nasal inferior wall, and the black arrows indicate increased FDG activity in the inferior wall consistent with an inflammatory process. B: The upper panel shows a septal perfusion defect and the red arrows indicate increased FDG activity in the inferior septum consistent with an inflammatory process. C: The white arrows indicate a moderate-size rest perfusion defect in the inferior wall, and the black arrows indicate increased FDG activity in the inferior wall consistent with an inflammatory process. FDG: 18F-fluorodeoxyglucose; PET: positron emission tomography

**Figure 5:**
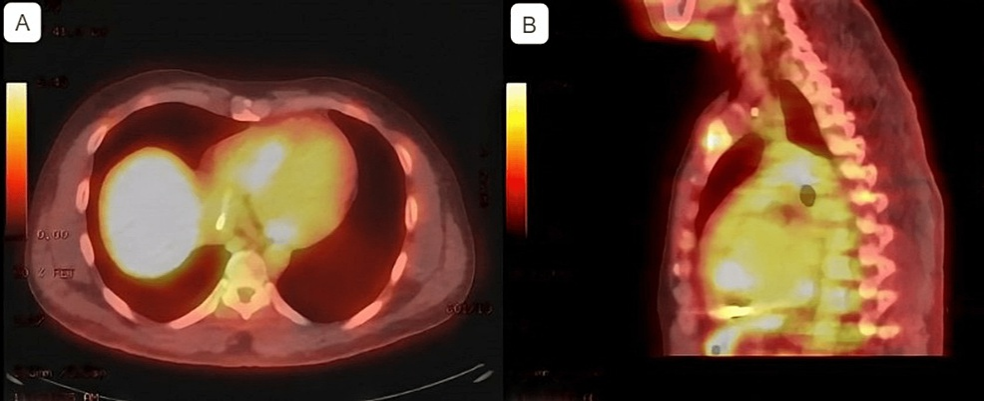
PET/CT scan showing a focal uptake of FDG in the inferior septum, indicating active inflammation A: Transverse section showing increased FDG uptake in the inferior septum B: Sagittal section showing increased FDG uptake in the inferior septum FDG: 18F-fluorodeoxyglucose; PET: positron emission tomography

To confirm the diagnosis of CS, PET with FDG was done and demonstrated severe perfusion defect in the basal segment of the inferior septum with FDG focal uptake in the inferior septum, indicating active inflammation and consistent with CS (Figures 4, 5).

To characterize the extent of the disease, full-body PET was done and showed no evidence of extracardiac involvement. During the patient’s evaluation, an empty sella turcica was incidentally noted on brain CT.

Diagnosis and treatment outcomes

Based on CMR findings that were further supported by PET with FDG, a diagnosis of isolated cardiac sarcoidosis (ICS) was made. Rheumatology was consulted regarding the treatment plan, and it was decided to start the patient on daily prednisone after checking viral hepatitis serology and TB Quantiferon. He was started on oral prednisone 80 mg once daily for 14 days, with a plan of decreasing the dose by 10 mg weekly until 40 mg once daily and then by 5 mg until 20 mg once daily. The patient was discharged with a notable decrease in the frequency of PVCs and given a follow-up appointment with rheumatology after three months.

## Discussion

Sarcoidosis is a multisystemic, immune-mediated inflammatory disease characterized by non-caseating granulomas involving virtually any organ in response to an unknown antigen [1,2]. Its manifestations are highly heterogenous, unpredictable, and largely dependent on the involved organs. Although the lungs and lymphatics are the most commonly affected, up to 30% of patients present with extrapulmonary infiltration, involving the skin, nervous system, musculoskeletal system, gastrointestinal tract, kidney, and heart [4,8]. In this article, we report a case of isolated cardiac sarcoidosis (ICS) in a 42-year-old male with non-ischemic cardiomyopathy and a remote history of anabolic steroid use presented as non-sustained polymorphic ventricular tachycardia.

We believe that this case is interesting due to the following reasons: 1) This case highlights the importance of maintaining a high index of clinical suspicion for CS and keeping it in mind when a young patient presents with bradycardia with atrioventricular block and episodes of non-sustained polymorphic ventricular tachycardia. Although sarcoidosis generally follows a benign course and might resolve spontaneously, cardiac involvement can complicate the patient’s clinical course with fatal arrhythmias, such as ventricular fibrillation, and heart failure [4,5,9]. Because of that, timely diagnosis and corticosteroid initiation are essential to mitigate CS-related morbidity and mortality. 2) Similar to amyloidosis, sarcoidosis is an infiltrative disease that classically causes restrictive cardiomyopathy [10]. Our patient has severe systolic dysfunction, with an EF of 30-35% based on echocardiogram and 27% based on CMR, and eccentric hypertrophy. 3) This case is rare as only 25% of CS patients have ICS [11]. 4) To the best of our knowledge, this might be the first reported case of ICS in the Kingdom of Saudi Arabia [12]. However, this case is limited by the absence of a histopathological diagnosis and the unavailability of a follow-up PET/CT with FDG after completing the treatment course.

Although the atrioventricular block is the most common presenting arrhythmia in patients with CS [13], there have been several reports of ventricular arrhythmia as the initial presentation of CS, particularly in patients with EF<25% [7,14-17]. To emphasize, more than half of CS cases with EF<25% have ventricular tachycardia within five years of CS diagnosis [6]. Our patient had an ICD implanted about a year ago, which can be life-saving as the risk of sudden cardiac death is estimated to be 9% and 34% in patients with normal EF and with EF<25% or prior history of ventricular arrhythmia, respectively [6].

Owing to the heterogeneous and unpredictable presentation of CS, and the fact that routine cardiac imaging, such as ECG and echocardiogram, cannot diagnose CS, it represents a highly challenging diagnosis, especially in the absence of systemic manifestations [18]. Endomyocardial biopsy was used to diagnose CS, but it is invasive, and its value is highly limited by its low sensitivity due to the patchy nature of sarcoidosis. Recently, CMR and PET with FDG have largely replaced endomyocardial biopsy [6]. The classical findings of PET/CT with FDG and CMR in CS are patchy uptake and late gadolinium enhancement that is mainly seen in the basal segments of the septum and lateral wall and usually in the mid-myocardium and sub-epicardium, respectively [2]. Our patient was diagnosed by CMR and PET with FDG, as these can reliably identify cardiac inflammation. Full body PET was done for our patient to identify the extent of the disease, and this can guide the treatment plan as patients with multiorgan involvement might benefit from immunosuppressants such as methotrexate in addition to corticosteroids. Finally, elevated serum ACE is a common finding that can be seen in roughly 30-80% of sarcoidosis cases, and its sensitivity ranges from 22-86% and specificity from 54-95% [10]. Our patient has a normal ACE level, which might be attributed to the fact that the ACE level was tested after prednisone initiation.

## Conclusions

ICS is an exceedingly rare form of extrapulmonary sarcoidosis. The recognition of ICS requires a high index of clinical suspicion and a proper interpretation of CMR and PET with FDG. Since the prognosis of ICS can be extremely poor, prompt diagnosis and glucocorticoid initiation are crucial to prevent CS-related complications such as heart failure and ventricular arrhythmias. This case highlights the importance of keeping CS in the differential diagnosis when a young patient presents with bradycardia with atrioventricular block and episodes of non-sustained polymorphic ventricular tachycardia.
